# Hyodeoxycholic acid ameliorates tacrolimus-induced diabetes by modulating the FXR-FGF15 axis

**DOI:** 10.3389/fphar.2026.1865237

**Published:** 2026-07-28

**Authors:** Nan Hu, Minyan Qian, Yanru Liu, Zhenwei Jiang, Caomei Xu, Wenting Zhang, Jingting Jiang

**Affiliations:** 1 Department of Pharmacy, The Third Affiliated Hospital of Soochow University/The First People’s Hospital of Changzhou, Changzhou, China; 2 Pediatric Central Laboratory, Affiliated Changzhou Children’s Hospital of Nantong University, Changzhou, China; 3 The Third Affiliated Hospital of Soochow University, Department of Tumor Biological Treatment, Changzhou, China

**Keywords:** bile acid metabolism, tacrolimus-induced diabetes mellitus, FXR-FGF15 axis, gut microbiota, hyodeoxycholic acid (HDCA), tacrolimus

## Abstract

**Backgroud:**

Immunosuppressant tacrolimus (TAC) induces glucose metabolism disorder and diabetes mellitus (DM) closely associated with intestinal microbiota dysbiosis and reduced bile acid levels, and this study aimed to explore the ameliorative effect and underlying mechanism of hyodeoxycholic acid (HDCA) on TAC-induced DM in rat models.

**Methods:**

We first verified the critical role of intestinal microbiota and bile acids in the pathogenesis of TAC-induced DM via antibiotic-induced gut microbiota depletion, then orally administered HDCA (100 mg/kg) to TAC-induced diabetic rats to evaluate its protective efficacy, detecting glycolipid metabolism indices, targeted bile acid omics, FXR/TGR5 protein expression, serum and ileal GLP/FGF15 levels, and mRNA levels of key metabolic genes including *Cyp7a1, Cyp27a1, Cyp7b1, Cyp8b1, Fgfr4, Creb1* and *Ppargc1a*.

**Results:**

Antibiotic treatment significantly reduced BSH-active gut microbiota such as *Bacteroides* and *Lactobacillus*, disrupting the serum bile acid pool and exacerbating diabetic symptoms by altering the gut microbiota-bile acid axis; while HDCA administration markedly improved glucose tolerance and reshaped bile acid profiles in TAC-induced diabetic rats, specifically lowering serum and fecal 12-OH/Non-12-OH BAs ratio via downregulating hepatic *Cyp7a1* and upregulating hepatic *Cyp8b1* expression. HDCA also suppressed enteroendocrine cell-derived GLP-1 secretion, decreased serum and ileal FGF15 levels, and reduced the expression of ileal FXR as well as hepatic Creb1 and Ppargc1a.

**Conclusions:**

Collectively, HDCA exerts a significant ameliorative effect on TACinduced DM by regulating metabolic enzymes and altering bile acid profiles, with the core mechanism involving inhibited enterohepatic FXR-FGF15 axis, increased GLP-1 secretion and suppressed hepatic gluconeogenesis, all of which play key regulatory roles in this protective process.

## 1 Introduction

Tacrolimus (TAC) is a calcineurin inhibitor (CNI) primarily used in solid organ transplantation to prevent rejection and treat autoimmune diseases (Tonshoff, 2020). It exerts its pharmacological effect by forming a pharmaco-immune complex with FK506-binding protein 12 (FKBP12), which prevents the dephosphorylation of calcineurin (CaN), thereby suppressing the immune system through the inhibition of T lymphocyte activation and proliferation (Azzi et al., 2013). Despite its efficacy, TAC treatment is associated with a high incidence of post-transplantation diabetes mellitus (PTDM); approximately 33.6% of non-diabetic transplant recipients develop PTDM or impaired fasting glucose within 6 months after transplantation (Jenssen et al., 2019). Although TAC is the first-line medication recommended by the Global Guidelines for the Improvement of Renal Disease, it carries a significant risk of inducing glucose metabolism abnormalities (Kanbay et al., 2024). Current studies suggest that TAC primarily causes direct damage to pancreatic islet cells or impairs insulin signaling, which is considered the main pathogenesis of tacrolimus-induced diabetes (Rodriguez-Rodriguez et al., 2021). In animal models of tacrolimus-induced diabetes, alterations in glucose metabolic parameters are associated with the deterioration of pancreatic islet β-cell function, resembling the pathophysiological mechanism of type 2 diabetes mellitus (T2DM) (Quintana-Perez et al., 2022). Antidiabetic drugs such as metformin and insulin are widely used in managing PTDM, but effective strategies to prevent TAC-induced diabetes mellitus in post-transplant patients are still lacking (Shivaswamy et al., 2016). Therefore, studying the pathogenesis of TAC-induced diabetes, identifying novel intervention targets, and discovering new drugs for diabetes management are crucial (Zhang et al., 2018).

Recent studies have indicated that TAC induces gut microbiota dysbiosis, characterized by a reduced abundance of short-chain fatty acid-producing bacteria such as *Lachnospira multipara*, along with an elevated *Firmicutes/Bacteroidetes* ratio (Han et al., 2019; Kunasol et al., 2025). Given the established role of gut microbiota in diabetes pathogenesis, such dysbiosis is closely associated with an increased risk of obesity and T2DM (Fan et al., 2021; Gurung et al., 2020). In clinical transplantation, the use of immunosuppressive regimens increases susceptibility to opportunistic infections, frequently necessitating antibiotic treatment (Roberts et al., 2021). Although antibiotics target pathogenic bacteria, they also disrupt intestinal flora homeostasis, potentially leading to various diseases. Clinical studies have shown that long-term exposure to certain antibiotics (such as cephalosporins and macrolides) may increase the risk of diabetes in patients (Davis et al., 2019). Several *in vivo* experiments have also demonstrated the impact of antibiotics on blood glucose regulation (Yabe et al., 2019; Zheng et al., 2019). Consequently, antibiotic-induced gut microbiota depletion models are widely employed to elucidate the causal role of microbial communities in disease development, including diabetes (Zhai et al., 2024). Notably, the intestinal microbiota, including genera like *Bacteroides*, *Lactobacillus*, and *Ruminococcus*, plays a critical role in bile acid metabolism (Guo et al., 2022; Jia et al., 2018). Bile acids (BAs), as key microbial-host co-metabolites, are involved in various metabolic processes, including energy and glucose homeostasis (Wu et al., 2021). In our previous study, we found that the alteration of intestinal flora in TAC-induced diabetic animals was closely correlated with bile acid levels. Specifically, the abundance of bile acid metabolizing genera such as *Ruminococcus* and *Roseburia* was significantly reduced. Additionally, fasting blood glucose (FBG) and insulin levels were correlated with the abundance of *Ruminococcus*, *Akkermansia*, *Roseburia*, and bile acid levels such as cholic acid (CA) and chenodeoxycholic acid (CDCA) (Jiang et al., 2024). These findings suggest that changes in gut flora composition and bile acid levels may directly contribute to the development of TAC-induced diabetes (Gurung et al., 2020; Huda et al., 2021).

Bile acids are the end products of cholesterol catabolism in the liver, with the majority of primary bile acids synthesized via the classical pathway, and a small proportion (approximately 6% of human bile acids) synthesized through the alternative pathway. Cholesterol generates CA and CDCA through the classical pathway mediated by cholesterol-7α-hydroxylase (CYP7A1), which regulates the size of the hepatic bile acid pool (Chiang et al., 2018). Cholesterol also generates CDCA through an alternative pathway mediated by sterol 27-hydroxylase (CYP27A1) and oxysterol 7α-hydroxylase (CYP7B1). While CYP7A1 regulates bile acid (BA) pool size, the composition of BAs depends on the activity of sterol-12α-hydroxylase (CYP8B1) (Chiang et al., 2020). Overexpression of the hepatic enzyme CYP7A1 in mice significantly increases the BA pool size and prevents high-fat diet-induced obesity, insulin resistance, and hepatic steatosis (Chiang et al., 2020; Li et al., 2010). Numerous studies have demonstrated that alterations in BA concentration and composition are closely associated with disease onset and progression (Fleishman et al., 2024).

The use of pig bile for treating “consuming thirst” (now known as diabetes mellitus) is documented in the Compendium of Materia Medica. As early as 1923, Windaus isolated hyocholic acid (HCA) and hyodeoxycholic acid (HDCA) from porcine bile. HCA and its derivatives comprise more than 80% of porcine BAs, whereas they constitute only 2%–4% of BAs in mice and humans. The biosynthesis pathways of HCA and HDCA in humans remain incompletely understood. However, emerging evidence suggests that CDCA can be converted to HCA via CYP3A4-mediated 6α-hydroxylation (Zheng et al., 2021a). Another study demonstrated that CYP3A4 is a major hepatic enzyme responsible for the 6α-hydroxylation of lithocholic acid (LCA) and CDCA, leading to the formation of HDCA and HCA-related intermediates. Subsequent oxidation and epimerization reactions mediated by intestinal microbiota may further contribute to HDCA production (Deo et al., 2008; Zheng et al., 2021a). Recently, it has been reported that HCA and its derivatives improve glucose homeostasis by promoting the production and secretion of GLP-1 via unique FXR and TGR5 signaling pathways (Zheng et al., 2021a). HDCA, a natural secondary hydrophilic BA, has been shown to exert protective effects against metabolic disorders and holds diagnostic and therapeutic potential in regulating glucose homeostasis and predicting the future risk of T2DM (Zheng et al., 2021b).

Recent studies have highlighted the critical role of BA, key endogenous steroid molecules, in regulating lipid, glucose, and energy metabolism, modulating inflammatory responses, and influencing the development of metabolic disorders such as obesity and diabetes (Jia et al., 2024; Li et al., 2024). BAs activate FXR and TGR5 to regulate glucose, lipid, and energy metabolism (Chiang et al., 2020). Specifically, FXR activation not only modulates BA metabolism but also influences glucolipid homeostasis (Xiang et al., 2023). In enteroendocrine L cells, BA-activated FXR inhibits GLP-1 secretion and reduces glucagon expression, providing a new molecular mechanism for the regulation of blood glucose by BAs (Trabelsi et al., 2015). Hepatic BAs-FXR signaling regulates blood glucose levels by reducing hepatic gluconeogenesis and promoting hepatic glycogen synthesis (Shapiro et al., 2018). The downstream target of BA-activated FXR, fibroblast growth factor 15 (FGF15), is also crucial for glucose and insulin homeostasis (Rajani et al., 2018). Interestingly, emerging evidence suggests that targeted inhibition of the intestinal FXR signaling pathway (such as blunting the ileal FXR-FGF15/19 axis) can paradoxically alleviate obesity, non-alcoholic fatty liver disease (NAFLD), and other metabolic disorders (Luo et al., 2021; Sun et al., 2021). Increasing evidence suggests that BAs regulate glycolipid metabolism through receptor binding, mediating the development of metabolic diseases (Jia et al., 2018; Li et al., 2022).

In this study, we aimed to investigate the ameliorative effects of HDCA on TAC-induced diabetes in a rat model, and to explore its underlying mechanisms by targeting the FXR-FGF15 axis in BA anabolic metabolism and associated downstream signaling molecules.

## 2 Materials and methods

### 2.1 Materials and reagents

Tacrolimus (TAC, P-JH210329, purity≥98%) was purchased from POPEYE (Shenzhen, China). Hyodeoxycholic acid (HDCA, A2206625, purity≥95%) was purchased from Aladdin (Shanghai, China). Ampicillin (A830931, purity≥98%), Neomycin sulfate (N799581, purity≥97%), Metronidazole (M813526, purity≥99%), and Vancomycin (V871983, USP) were purchased from Macklin (Shanghai, China). NR1H4 polyclonal antibody (anti-FXR, 25055-1-AP), Insulin polyclonal antibody (anti-INS, 15848-1-AP) were purchased from Proteintech (Wuhan, China), TGR5 polyclonal antibody (anti-TGR5, YT4636) was purchased from Immunoway (Texas, United States). BAs standards were from Sigma-Aldrich (Shanghai, China), including CA, CDCA, LCA, HCA, HDCA, allocholic acid (ACA), isolithocholic acid (isoLCA), deoxycholic acid (DCA), isodeoxycholic acid (isoDCA), ursodeoxycholic acid (UDCA), glycocholic acid (GCA), taurocholic acid (TCA), glycodesoxycholic acid (GDCA), glycoursodeoxycholic acid (GUDCA), glycochenodeoxycholic acid (GCDCA), glycolithocholic acid (GLCA), glycohyodeoxycholic acid (GHDCA), taurodeoxycholic acid (TDCA), taurolithocholic acid (TLCA), taurochenodeoxycholic acid (TCDCA), taurohyodeoxycholic acid (THDCA), tauroursodeoxycholic acid (TUDCA), norcholic acid (NorCA), normethyodeoxycholic acid (NorUDCA), nordeoxycholic acid (NorDCA), 7-ketodeoxycholic acid (7-KetoDCA), 7-ketolithocholic acid (7-KetoLCA), 12-ketolithocholic acid (12-KetoLCA), 7,12-diketolithocholic acid (7,12-DKLCA), glycocholic acid-d5 (GCA-d5), chenodeoxycholic acid-d4 (CDCA-d4), deoxycholic acid-d4 (DCA-d4), and lithocholic acid-d4 (LCA-d4). Acetate, propionate, butyrate, and pentanoic acid were purchased from Aladdin (Shanghai, China). All the other reagents applied in this study are of analytical grade.

### 2.2 Animal and experimental design

Male Sprague-Dawley (SD) rats (6–8 weeks old, approximately 200 g) were obtained from Changzhou Cavins Laboratory Animal Co. Ltd. (License No. SCXK (Su) 2021–0,013). All rats were housed in a specific pathogen-free (SPF) facility (25 °C), with unrestricted access to water and food, and maintained on a 12-h light/dark cycle. Prior to the experiment, the rats were acclimatized for 1 week. The animal protocol adhered to institutional guidelines for the care and use of laboratory animals and was approved by the Ethics Committee of the Third Affiliated Hospital of Soochow University (Approval No. 2021151).

Antibiotic intervention: After acclimatization, rats were randomly assigned to three groups (n = 6 per group): (1) control-A (CON-A) group: intraperitoneal injection of an equal volume of saline; (2) tacrolimus-A (TAC-A) group: intraperitoneal injection of 3 mg/kg/day TAC; and (3) tacrolimus combined with antibiotics (TAC + ABX) group: intraperitoneal injection of 3 mg/kg/day TAC, followed by gavage with antibiotics (ampicillin, neomycin sulfate, and metronidazole at 100 mg/kg/12 h; vancomycin at 50 mg/kg/12 h).

Hyodeoxycholic acid intervention: Following the acclimatization period, rats were randomly assigned to three groups (n = 6 per group): (1) control (CON) group: intraperitoneal injection of an equal volume of saline; (2) tacrolimus (TAC) group: intraperitoneal injection of 3 mg/kg/day TAC; and (3) tacrolimus combined with hyodeoxycholic acid (TAC + HDCA) group: intraperitoneal injection of 3 mg/kg/day TAC, followed by 100 mg/kg/day of hyodeoxycholic acid administered by gavage.

All interventions were continued for 10 weeks. Body weight was recorded weekly during the experiment. Fresh fecal samples were collected from each rat 1 week before the conclusion of the experiment and stored at −80 °C until analysis. At the end of the experiment, all rats were anesthetized with Zoletil® 50 (50 mg/kg; Virbac, Carros, France), and samples of serum, liver, pancreas, ileum, and colon were collected and stored at −80 °C for further analysis.

### 2.3 Fasting blood glucose (FBG) and oral glucose tolerance test (OGTT)

An OGTT was performed on rats at week 10. The rats were placed in clean cages and fasted with free access to water for 12 h. FBG was measured from the tail vein using a glucometer (Roche, Basel, Switzerland). Subsequently, a 20% glucose solution (2 g/kg) was administered by gavage, and blood glucose levels were measured at 30, 60, 90, and 150 min post-administration.

### 2.4 Histological analysis and immunohistochemistry

Rat pancreases were collected and fixed in 4% paraformaldehyde, then embedded in paraffin. Paraffin sections (4 μm) were stained with hematoxylin and eosin (HE), dehydrated, sealed, and observed under a light microscope (Nikon, Tokyo, Japan) to assess histopathological changes.

For immunohistochemical analysis, paraffin sections were deparaffinized and antigenically repaired, followed by incubation in 3% hydrogen peroxide, rinsing in PBS, and drying before being blocked in 5% BSA. Sections were then incubated overnight at 4 °C with an insulin-specific antibody diluted in the blocking solution. After washing the sections four times with TBST, sections were incubated with the corresponding enzyme-labeled secondary antibody. Following rinsing, the sections were incubated with freshly prepared DAB solution and re-stained with hematoxylin. Finally, the sections were dehydrated, sealed with neutral resin, and observed under a light microscope (Nikon, Tokyo, Japan) for image acquisition.

### 2.5 Enzyme-linked immunosorbent assay (ELISA)

Fasting serum insulin (FSI), glucagon-like peptide-1 (GLP-1), and FGF15 levels were measured using an ELISA kit (Bioswamp, Wuhan, China) according to the manufacturer’s instructions. ELISA plates were analyzed at 450 nm using a SpectraMax iD3 enzyme reader (Molecular Devices, San Jose, California, United States), and the mean optical density (OD) values were calculated.

### 2.6 16S rRNA gene sequencing

Microbial diversity sequencing: Total DNA of fecal microorganisms was extracted using the E. Z.N.A.® soil DNA kit (Omega Bio-tek, Norcross, GA, United States), and the extracted genomic DNA was separated by electrophoresis on a 1% agarose gel. DNA concentration and purity were assessed using a NanoDrop 2000 (Thermo Fisher Scientific, MA, United States). The V3-V4 region of the 16S rRNA gene was performed using primers 338F (5ʹ-ACT​CCT​ACG​GGG​GAG​GCA​GC AG-3ʹ) and 806R (5ʹ-GGACTACHVGGGTWTCTAAT-3ʹ). PCR products from the same samples were pooled and recovered on a 2% agarose gel. The recovered products were purified using the AxyPrep DNA Gel Extraction Kit (Axygen Biosciences, CA, United States). Library construction was carried out using the NEXTFLEX Rapid DNA-Seq Kit, followed by double-exponential amplification and sequencing on the Illumina MiSeq PE300 platform to assess gut microbiota structure.

Bioinformatics processing: Species taxonomic annotation of OTU representative sequences was performed using the RDP classifier based on the SILVA 16S rRNA (Release 138, https://www.arbsilva.de/documentation/release138/) database. QIIME (Version 1.6.0) software was used to calculate the ACE, Chao1, Shannon, and Simpson indices to analyze differences between groups in the Alpha diversity index. Principal coordinates analysis (PCoA) was performed to assess Beta diversity, and analysis of variance (ANOVA) was used to analyze group differences. The Kruskal–Wallis test and Linear discriminant analysis effect size (LefSe) were used to analyze group differences, with linear discriminant analysis (LDA) values greater than 4 considered statistically significant.

### 2.7 Serum and fecal BAs analysis

Serum BAs were determined using high-performance liquid chromatography tandem mass spectrometry (HPLC-MS/MS) as previously described (Sampson et al., 2016). Fecal BAs were determined as follows: fecal samples were homogenized in methanol (9 mL per Gram of fecal sample) and sonicated for 30 min, followed by centrifugation at 16, 400 rpm/min for 20 min. A 50 μL aliquot of the supernatant was mixed with 20 μL of internal standard solution (containing GCA-d5, CDCA-d4, DCA-d5, GCDCA-d7, and LCA-d4 at 100 ng/mL each) and 200 μL of acetonitrile. After vortexing for 30 s and centrifugation at 16, 400 rpm/min for 10 min, the supernatant was diluted with an equal volume of ultrapure water and transferred to a glass vial for HPLC-MS/MS analysis.

### 2.8 Quantitative real-time PCR (RT-PCR)

Total RNA was extracted using the E. Z.N.A.® HP Total RNA Kit (Omega Bio-Tek, Guangzhou, China) according to the manufacturer’s instructions. RNA concentration and purity were assessed using an Ultra-Micro Visible UV Spectrophotometer ND5000 (BioTeke, Beijing, China). cDNA was synthesized using the HiScript Q RT SuperMix for qPCR (+gDNA wiper) Reverse Transcription Kit (Vazyme, Nanjing, China), and the reverse transcription conditions were 50 °C for 15 min and 80 °C for 2 min using a LineGene9600plus (Bioer Technology, Hangzhou, China). PCR amplification was performed using ChamQ SYBR Color qPCR Master Mix (2X) (Vazyme, Nanjing, China) under the following conditions: 95 °C for 10 min; 40 cycles of amplification (95 °C for 30 s, 56 °C for 30 s, 72 °C for 40 s). The relative gene expression was determined using the 2^−ΔΔCt^ method. All primer sequences are provided in Table 1.

**TABLE 1:** Primer sequences for RT-PCR.

Gene	Forward primer (5ʹ-3ʹ)	Reverse primer (5ʹ-3ʹ)
*Cyp27a1*	TGT​TCG​ACA​CAT​CCT​GAT​TG	GAG​GCA​GAA​CTC​CAG​CTT​TG
*Cyp7a1*	AAG​GAG​AAG​GAA​AGC​TGG​TG	ACC​ATG​CTT​CCT​TTG​ATT​AG
*Cyp7b1*	TCT​CTC​CCT​ACT​AGG​CCT​TC	GCC​AAG​ATA​AGG​AAT​CCA​AC
*Cyp8B1*	ATG​TTT​GAA​TTC​CTG​AAG​GG	TCT​GCG​TGC​TCT​TAA​TGA​TG
*Fgfr4*	GGC​TTT​GTT​GAG​CAT​CTT​TC	CAG​TCA​ACA​CCT​GCT​CTT​GC
*Ppargc1a*	TTG​CCC​AGA​TCT​TCC​TGA​AC	TAC​ACC​ACT​TCA​ATC​CAC​CC
*Creb1*	GCC​ACA​GAT​TGC​CAC​ATT​AG	GAC​TGA​ATA​ACT​GAT​GGC​TG
*Actb*	CCC​ATC​TAT​GAG​GGT​TAC​GC	TTT​AAT​GTC​ACG​CAC​GAT​TTC

### 2.9 Western blot (WB)

Rat ileum tissues were collected, and total tissue proteins were extracted using a Membrane and Cytosol Protein Extraction Kit (Beyotime, P0033-1, Shanghai, China). Protein concentration was subsequently determined using a BCA assay kit (Beyotime, P0010, Shanghai, China). Extracted intracellular proteins (30 μg) were separated by 10% SDS-PAGE and transferred to polyvinylidene difluoride (PVDF) membranes according to the standard wet transfer protocol. The membranes were blocked with 5% skimmed milk powder for 1 h at room temperature, incubated with the appropriate primary antibody (NR1H4 polyclonal antibody or TGR5 polyclonal antibody) overnight at 4 °C, and washed four times with TBST. The membranes were then incubated with HRP-labeled goat anti-rabbit IgG or goat anti-mouse IgG for 1 h at room temperature and washed four times with TBST. The membranes were visualized using a multifunctional chemiluminescence imaging system (Tanon 4600, Shanghai, China), and internal controls from the same blot were detected using a β-actin antibody.

### 2.10 Data analysis

The data were processed using GraphPad Prism 9 (Version 9.2.0, San Diego, United States). All experimental results are expressed as mean ± SD. Normally distributed data were analyzed using one-way analysis of variance (ANOVA) followed by Tukey’s *post hoc* test, whereas non-normally distributed data were analyzed using the Kruskal–Wallis test followed by Dunn’s multiple-comparison test. Correlation analysis was performed using the Pearson correlation test. Differences were considered statistically significant when *P* < 0.05.

## 3 Results

### 3.1 Effect of antibiotics on tacrolimus-induced diabetes mellitus

**FIGURE 1:**
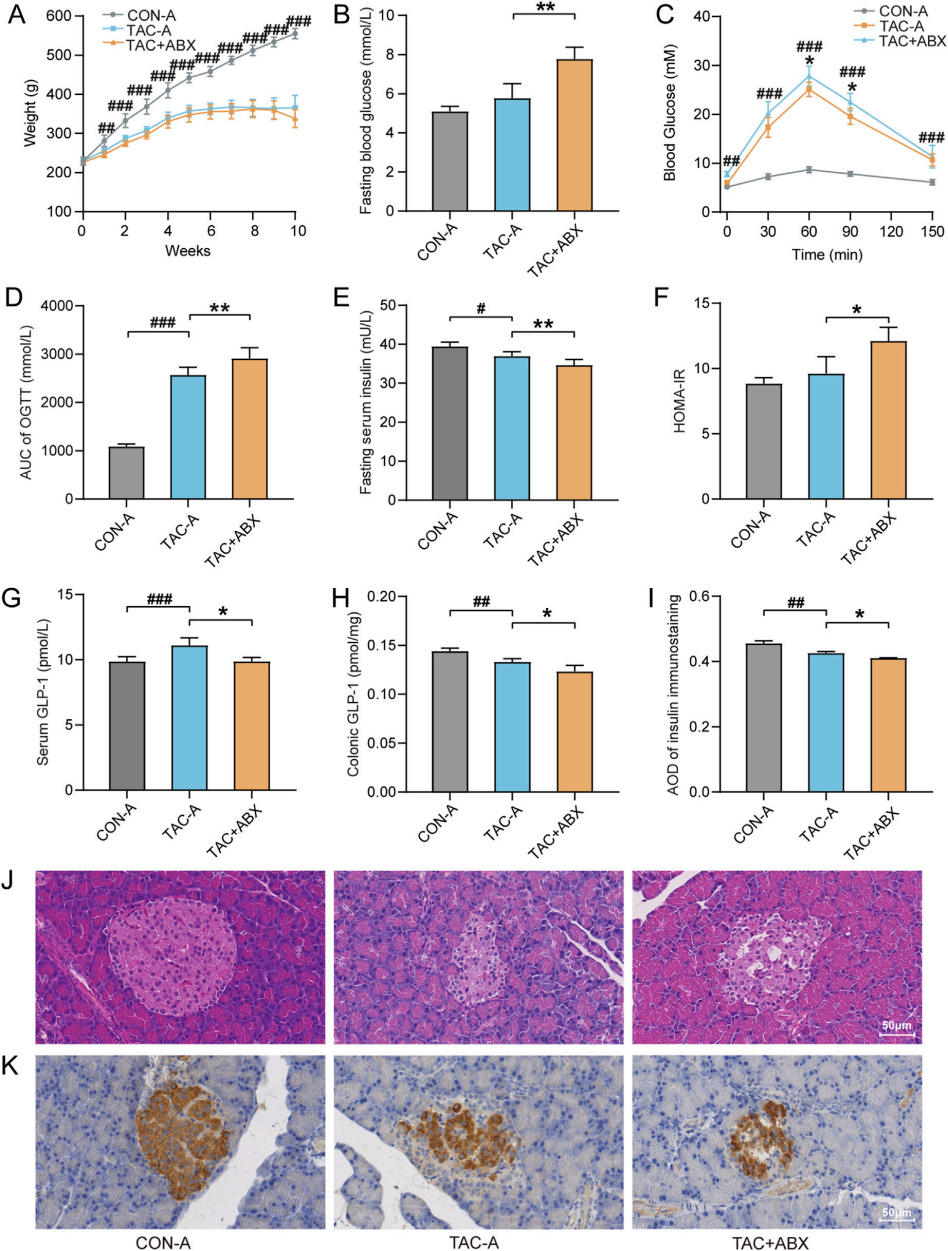
Effects of antibiotic-mediated microbiota depletion on glucose metabolism, GLP-1 secretion, insulin resistance, and pancreatic β-cell function in tacrolimus-induced diabetic rats. **(A)** Body weight. **(B)** Fasting blood glucose (FBG). **(C)** Oral Glucose Tolerance Test (OGTT). **(D)** AUC of OGTT. **(E)** Fasting serum insulin (FSI). **(F)** Homeostatic model assessment of insulin resistance (HOMA-IR). **(G)** Serum glucagon-like peptide-1 (GLP-1). **(H)** Colon GLP-1. **(I)** Average optical density (AOD) for insulin immunostaining in pancreatic islets. **(J)** Pancreatic histopathology (×400 magnification). Scale bar, 50 μm. **(K)** Representative immunohisto-chemistry images of insulin (×400 magnification). Scale bar, 50 μm. Data are presented as the mean ± SD. Statistical significance was determined by ANOVA test followed by Tukey’s *post hoc* test (n = 6 per group). Significant differences are denoted as ^#^ *P* < 0.05, ^##^ *P* < 0.01, and ^###^ *P* < 0.001 for TAC-A versus CON-A, and as ^*^ *P* < 0.05, ^**^ *P* < 0.01, and ^***^ *P* < 0.001 for TAC + ABX versus TAC-A group.

To determine the effect of intestinal flora on TAC-induced diabetes, rats were divided into the CON-A, TAC-A, and TAC + ABX groups. The intestinal flora was depleted by administering a mixture of antibiotics (ampicillin, neomycin sulfate, metronidazole, and vancomycin) by gavage to the rats in the TAC + ABX group. After 10 weeks of administration, the body weight of rats in both the TAC-A and TAC + ABX groups was significantly lower than that of the CON-A group (Figure 1A). FBG was significantly higher in the TAC + ABX group compared to both the CON-A and TAC-A groups (Figure 1B). Further oral OGTT experiments showed that blood glucose levels in the TAC-A and TAC + ABX groups significantly increased at 30, 60, 90, and 150 min after glucose loading compared to the CON-A group (Figure 1C). Meanwhile, the OGTT-AUC was significantly higher in the TAC + ABX group than in the CON-A and TAC-A groups (Figure 1D).

Compared with the CON-A group, FSI levels were significantly lower in both the TAC-A and TAC + ABX groups, with FSI levels further decreased in the TAC + ABX group compared to the TAC-A group (Figure 1E). Although not statistically significant, the HOMA-IR index was elevated in the TAC-A group compared with the CON-A group, however, antibiotic intervention in the TAC + ABX group led to a significant increase in HOMA-IR index (Figure 1F). Since insulin secretion is regulated by GLP-1, we subsequently examined GLP-1 levels in serum and colon. The results showed that both serum and colonic GLP-1 levels were significantly reduced in the TAC-A and TAC + ABX groups compared to the CON-A group. Additionally, serum and colonic GLP-1 levels were further reduced in the TAC + ABX group compared to the TAC-A group (Figures 1G,H).

HE staining revealed that, compared with the CON-A group, the TAC-A group exhibited a decreased number of pancreatic islet cells, smaller and irregular cell clusters, cytoplasmic vacuolization, uneven nuclear staining, and blurred boundaries between islets and surrounding tissues.ABX intervention further aggravated the damage to pancreatic islet β-cell, with obvious vacuolization observed (Figure 1J). Immunohistochemical analysis showed that the insulin-stained area was significantly reduced in the TAC-A group compared with the CON-A group. Furthermore, the insulin staining area was further reduced in the TAC + ABX group compared with the TAC-A group (Figures 1I,K).

### 3.2 Effect of antibiotics on intestinal flora composition and metabolite BAs in tacrolimus-induced diabetic rats

**FIGURE 2:**
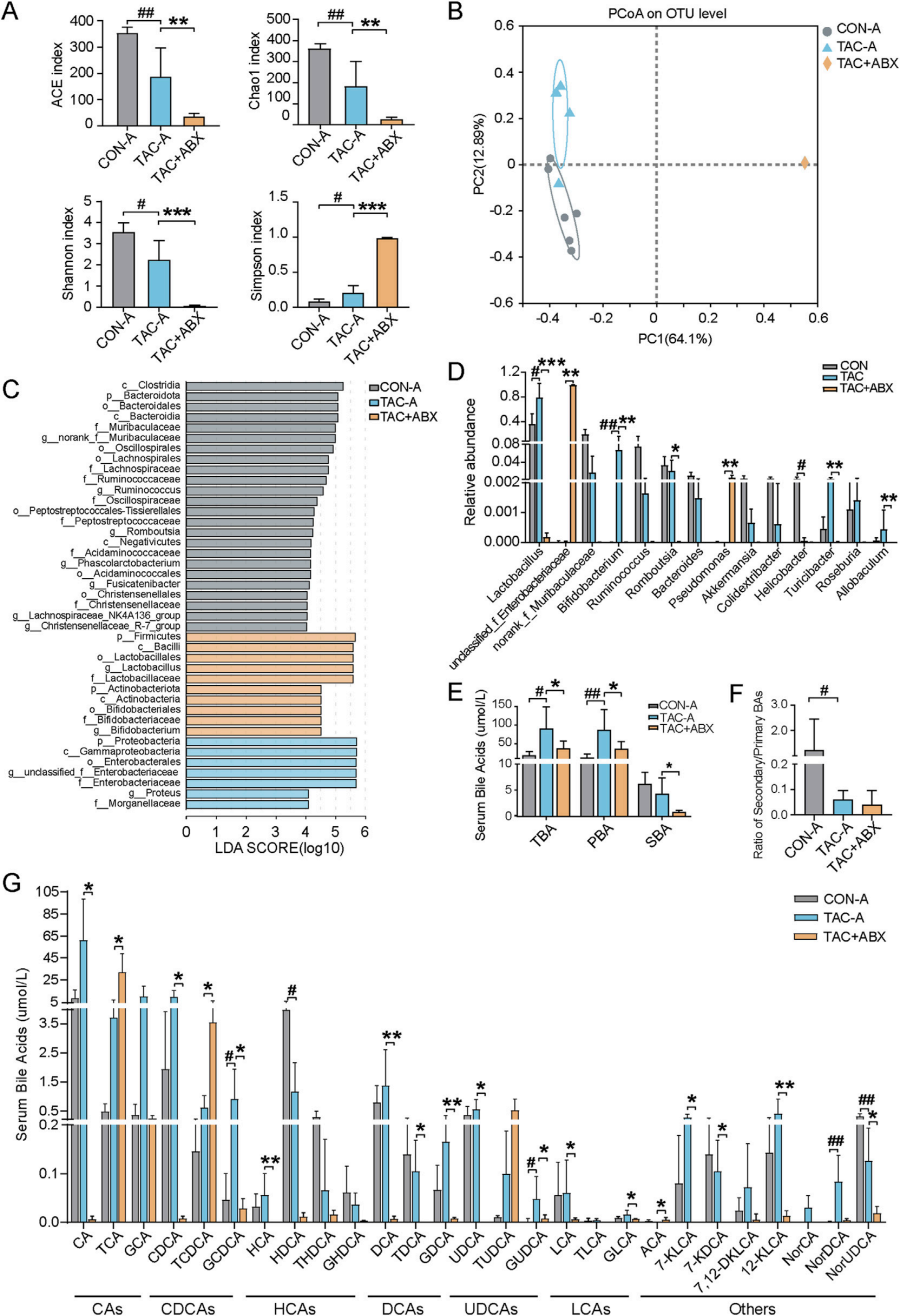
Antibiotic-induced gut microbiota depletion aggravates serum bile acid metabolic disturbances in tacrolimus-induced diabetic rats. **(A)** Intestinal flora alpha-diversity. **(B)** Principal co-ordinates analysis (PCoA). **(C)** Multilevel taxonomic linear discriminant analysis effect size (LEfSe) analysis. **(D)** Relative abundance of intestinal flora at genus level. **(E)** Serum levels of total bile acids (TBA), primary bile acids (PBA), and secondary bile acids (SBA) in different experimental groups. **(F)** Ratio of serum SBA to PBA. **(G)** Concentrations of individual bile acid species in serum. Data are presented as the mean ± SD. Normally distributed data were analyzed using one-way ANOVA followed by Tukey’s *post hoc* test; Non-normally distributed data were analyzed using the Kruskal–Wallis test followed by Dunn’s multiple-comparison test For fecal analyses, n = 6 (CON), n = 5 (TAC; one fecal sample was unavailable), and n = 6 (TAC+ABX). For bile acid analyses, n = 6 per group. Significant differences are denoted as ^#^ *P* < 0.05, ^##^ *P* < 0.01, and ^###^ *P* < 0.001 for TAC-A versus CON-A group, and as ^*^ *P* < 0.05, ^**^ *P* < 0.01, and ^***^ *P* < 0.001 for TAC + ABX versus TAC-A group.

To further clarify the role of gut microbiota, 16S rRNA sequencing was performed. The results showed that the ACE, Chao1, and Shannon indices decreased in the TAC-A group compared to the CON-A group, while the Simpson index was significantly lower. ABX intervention further affected the α-diversity of intestinal flora in TAC-induced diabetic rats. The ACE, Chao1, and Shannon indices were significantly lower in the TAC + ABX group compared to the TAC-A group, whereas the Simpson index was significantly higher in the TAC-A group (Figure 2A). Principal coordinates analysis (PCoA) revealed significant separation among the three groups, indicating that the composition of the intestinal flora differed significantly across the groups (Figure 2B). LEfSe analysis (LDA score>4) identified that the genera *c_Clostridia*, *p_Bacteroidota*, *o_Bacteroidales*, and *c_Bacteroidia* were significantly enriched in the CON-A group, while *p_Firmicutes*, *c_Bacilli*, *o_Lactobacillales*, and *f_Lactobacillaceae* were significantly enriched in the TAC-A group. In contrast, *p_Proteobacteria*, *f_Enterobacteriaceae*, *o_Enterobacterales*, and *g_Proteus* were significantly enriched in the TAC + ABX group (Figure 2C). Further analysis at the genus level revealed that *Lactobacillus* and *Bifidobacterium* were significantly enriched in the TAC-A group, while Helicobacter showed a decreasing trend compared to the CON-A group. ABX further exacerbated the changes in gut flora at the genus level. In the TAC + ABX group, *Lactobacillus, Bifidobacterium, Rumboutsia, Turicibacter*, and *Allobaculum* had significantly lower relative abundances compared to the TAC-A groups. In contrast, the relative abundance of *g_unclassified_f_Enterobacteriaceae* and *Pseudomonas*, which belong to *Proteobacteria* (phylum), was significantly higher in the TAC + ABX group compared to the TAC-A groups (Figure 2D).

BAs are important secondary metabolites of the gut microbiota, and it has been shown that antibiotic-induced depletion of the microbiome can disrupt metabolic homeostasis by altering the BA pool (Ussar et al., 2015). Therefore, we first analyzed the levels of total BAs (TBA), primary BAs (PBA), secondary BAs (SBA), and the ratio of SBA to PBA (SBA/PBA). We found that TAC significantly elevated TBA and PBA levels, while the SBA/PBA ratio was significantly reduced compared to the CON-A group. Antibiotic intervention restored TBA and PBA levels, further reducing SBA levels, whereas the SBA/PBA ratio was not significantly different from that of the TAC-A group (Figures 2E,F). Further analysis of serum BAs revealed significantly lower levels of HDCA and NorUDCA, and significantly higher levels of GCDCA, GUDCA, and NorDCA in the TAC-A group compared with the CON-A group. Notably, the levels of CA, CDCA, GCDCA, HCA, DCA, TDCA, GDCA, UDCA, GUDCA, LCA, GLCA, and NorUDCA were significantly reduced following antibiotic intervention compared to the TAC-A group, while the levels of TCA, TCDCA, and ACA were further elevated (Figure 2G). Among all analyzed BAs, GCDCA, NorUDCA, and GUDCA were significantly altered across all three groups. These results indicate that antibiotic-induced depletion of the microbiome exacerbates the dysregulation of serum BAs in TAC-induced diabetic rats.

**FIGURE 3:**
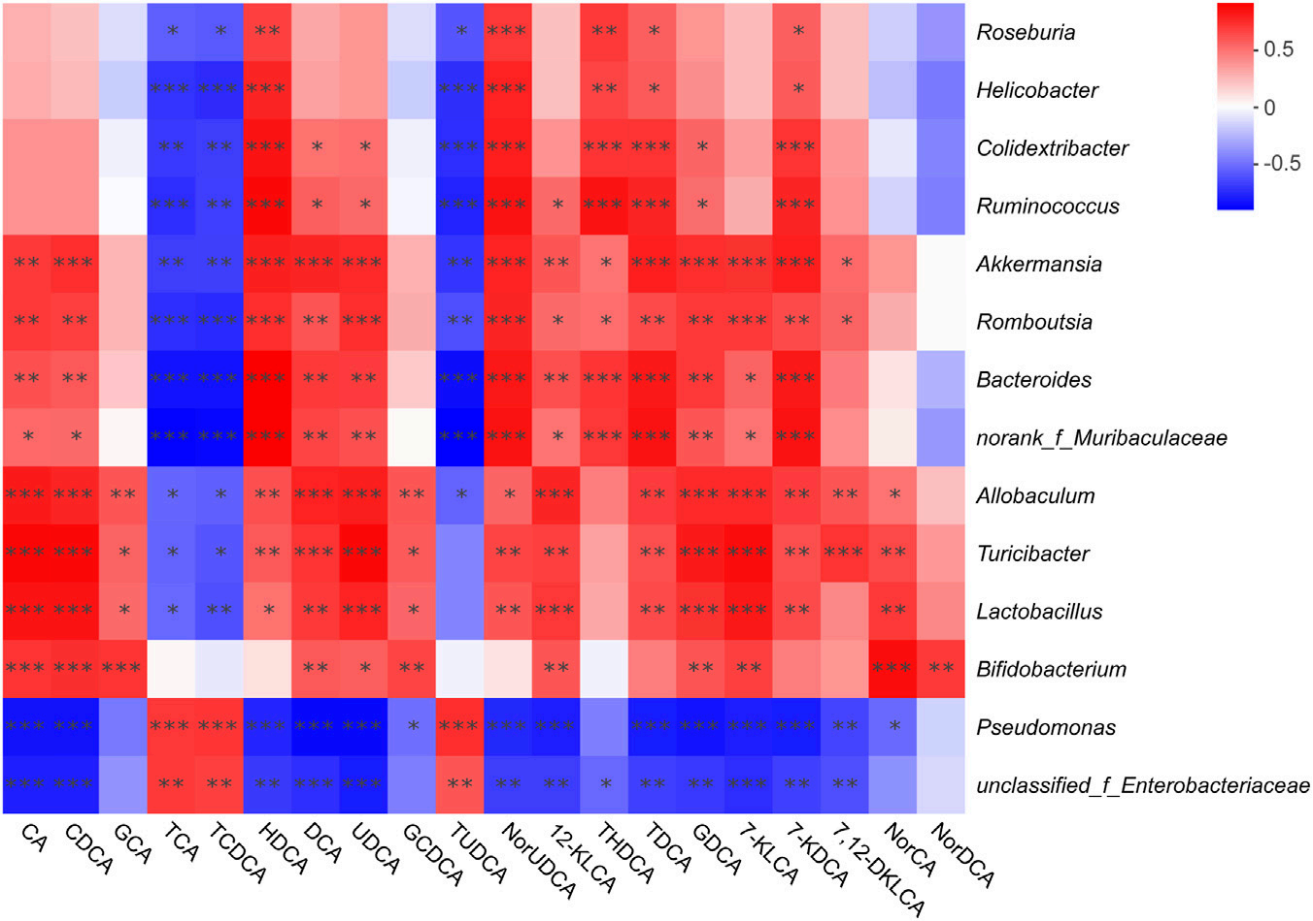
Correlation analysis between gut microbiota and serum bile acid profiles. A heatmap demonstrating the correlations between the relative abundance of specific bacterial genera and the concentrations of individual bile acids. The color gradient represents the correlation coefficient, where red indicates a positive correlation and blue indicates a negative correlation. Color intensity reflects the strength of the association. Statistical significance is denoted by asterisks (^*^ *P* < 0.05, ^**^ *P* < 0.01, ^***^ *P* < 0.001).

To elucidate the specific host-microbe interactions shaping BA pool, we performed a correlation analysis between the relative abundance of gut bacterial genera and BA profiles (Figure 3). The heatmap revealed that *Akkermansia*, *Bacteroides*, *Romboutsia*, *Ruminococcus*, *Colidextribacter*, and *norank_f_Muribaculaceae* all exhibited strong positive correlations with HDCA levels. This core bacterial consortium also showed similar positive correlations with other secondary bile acids (such as DCA and UDCA). In contrast, HDCA exhibited a highly significant negative correlation with potential pathobiont taxa, specifically *Pseudomonas* and *unclassified_f_Enterobacteriaceae*. Interestingly, *unclassified_f_Enterobacteriaceae and Pseudomonas* were strongly and positively correlated with taurine-conjugated primary bile acids (TCA, TCDCA, and TUDCA), while displaying negative correlations with almost all other BAs, including HDCA. Taken together, these data strongly indicate that the luminal enrichment of HDCA is closely associated with a specific, potentially beneficial microbial signature, which is characterized by the reduction of taurine-conjugated bile acids and the suppression of taxa such as *Pseudomonas* and *Enterobacteriaceae*.

### 3.3 Hyodeoxycholic acid improves tacrolimus-induced diabetes mellitus in rats

**FIGURE 4:**
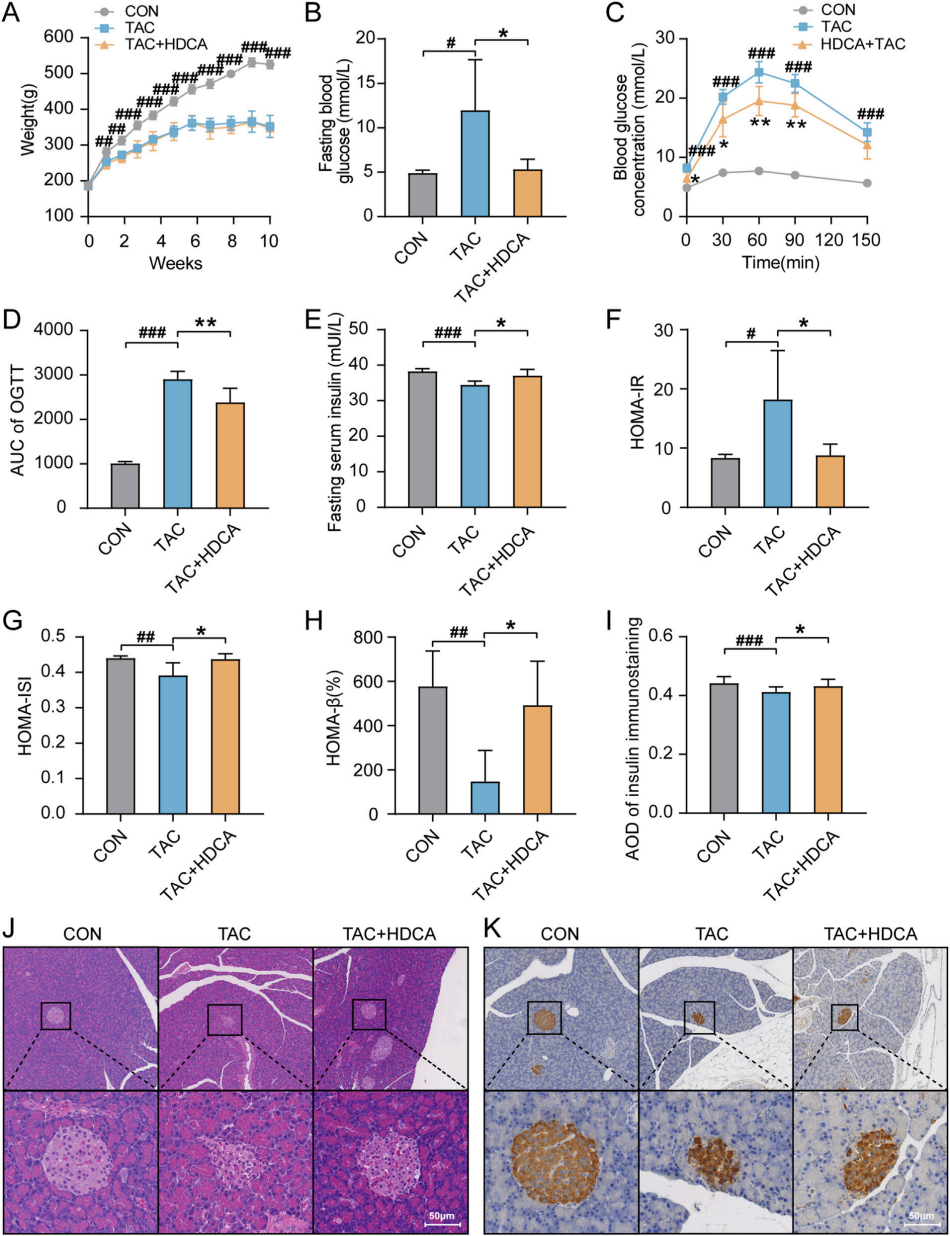
Effects of HDCA on metabolic phenotype, glucose homeostasis, and pancreatic histology in tacrolimus-induced diabetic rats. **(A)** Body weight. **(B)** FBG. **(C)** OGTT. **(D)** AUC of OGTT. **(E)** FSI. **(F)** HOMA-IR. **(G)** HOMA-ISI. **(H)** HOMA-β. **(I)** Average optical density (AOD) for insulin immunostaining in pancreatic islets. **(J)** Pancreatic histopathology (×400 magnification). Scale bar, 50 μm. **(K)** Representative immunohisto-chemistry images of insulin (×400 magnification). Scale bars, 50 μm. Data are presented as the mean ± SD. Statistical significance was determined by ANOVA test followed by Tukey’s *post hoc* test (n = 6 per group). Significant differences are denoted as ^#^ *P* < 0.05, ^##^ *P* < 0.01, and ^###^ *P* < 0.001 for TAC versus CON group, and as ^*^ *P* < 0.05, ^**^ *P* < 0.01, and ^***^ *P* < 0.001 for TAC + HDCA versus TAC group.

Compared with the CON group, the body weights of rats in both the TAC and TAC + HDCA groups were significantly lower, however, no significant difference in body weight was observed between the TAC and TAC + HDCA groups (Figure 4A). The results from FBG and OGTT experiments (Figures 4B,D) showed that, compared with the CON group, the rats in the TAC group exhibited significantly higher FBG levels and glucose levels at each time point after glucose stimulation, whereas in the TAC + HDCA group, FBG and glucose levels at each time point were significantly lower than those in the TAC group. The results of ELISA (Figure 4E) showed that FSI levels in the TAC group were significantly lower compared to the CON group, whereas FSI levels in the TAC + HDCA group were significantly higher than those in the TAC group. Calculation of the HOMA indices (Figures 4F–H) revealed that rats in the TAC group had significantly higher HOMA-IR indices and significantly lower HOMA-ISI/β indices compared to the CON group, whereas the TAC + HDCA group had significantly lower HOMA-IR indices and significantly higher HOMA-ISI/β indices than the TAC group. These results suggested that HDCA could improve the impaired glucose tolerance and insulin resistance induced by TAC.

HE staining of the pancreas (Figure 4J) showed that, compared with the CON group, the islet cells in the TAC group were reduced, and obvious vacuolization was observed. The islet cell clusters in the TAC + HDCA group were more regular in shape, with denser nuclei and clearer nucleoli than those in the TAC group. Insulin immunohistochemistry (Figures 4I,K) showed that, compared with the CON group, the islet area was smaller, and the intensity of insulin staining in the islets was lower in the TAC group. The intensity of insulin staining in the TAC + HDCA group was significantly higher than that in the TAC group. These results suggest that HDCA can improve the damage caused by TAC to islet cells and enhance insulin secretion.

### 3.4 Effect of hyodeoxycholic acid on serum and fecal BA levels in tacrolimus-induced diabetic rats

**FIGURE 5:**
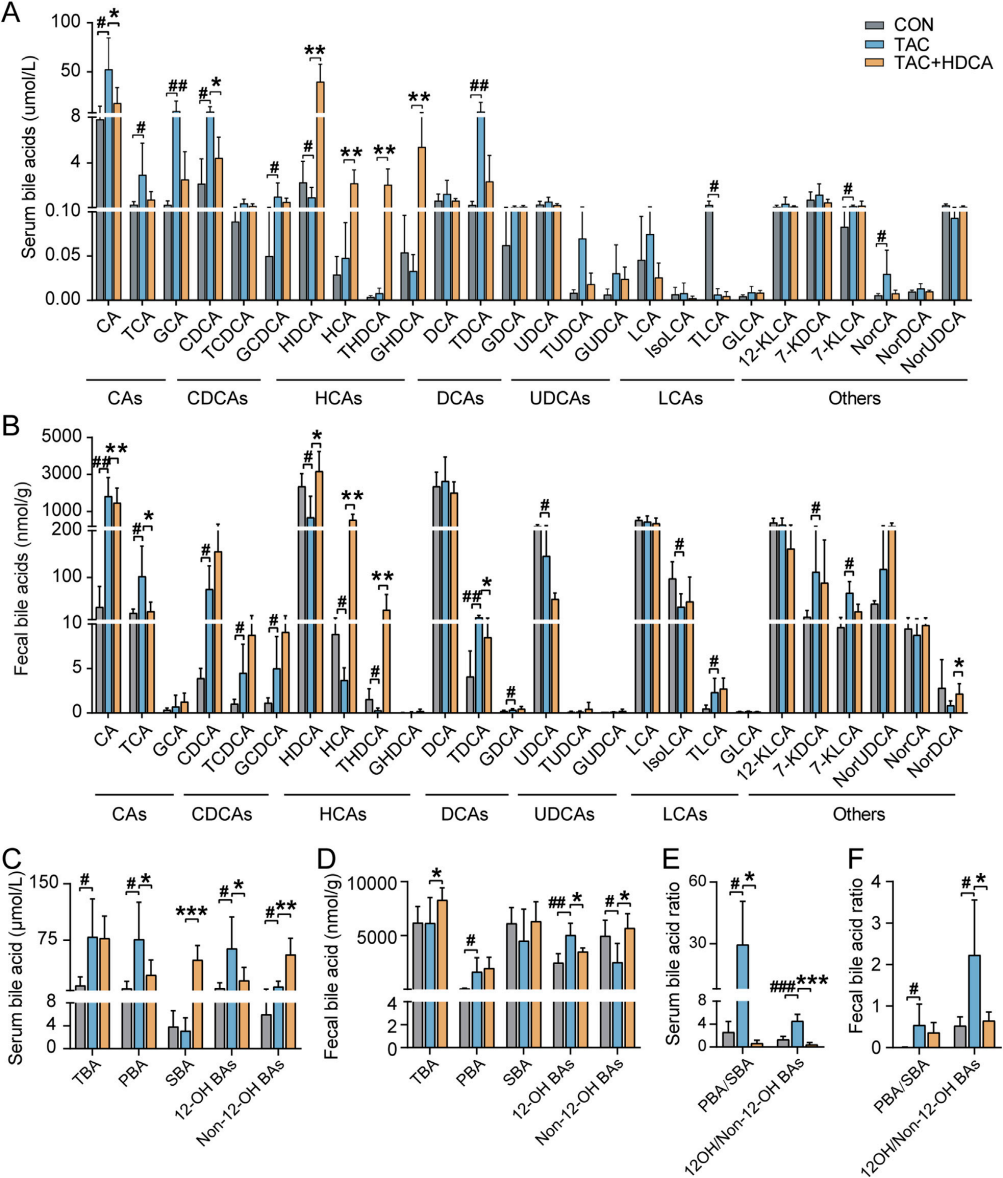
Effects of HDCA intervention on serum and fecal bile acid profiles in tacrolimus-induced diabetic rats **(A,B)** Concentrations of individual bile acid species in serum **(A)** and feces **(B)**. **(C,D)** Bile acid composition in serum **(C)** and feces **(D)**, including TBA, PBA, SBA, 12α-hydroxylated (12-OH) BAs, and Non-12-OH BAs **(E,F)** Bile acid ratios in serum **(E)** and feces **(F)**, including the SBA/PBA ratio and the 12-OH/Non-12-OH BAs ratio. Data are presented as the mean ± SD. Normally distributed data were analyzed using one-way ANOVA followed by Tukey’s *post hoc* test; Non-normally distributed data were analyzed using the Kruskal–Wallis test followed by Dunn’s multiple-comparison test (n = 6 per group). Significant differences are denoted as ^#^ *P* < 0.05, ^##^ *P* < 0.01, and ^###^ *P* < 0.001 for TAC versus CON group, and as ^*^ *P* < 0.05, ^**^ *P* < 0.01, and ^***^ *P* < 0.001 for TAC + HDCA versus TAC group.

To observe the effect of HDCA on BA levels in TAC-induced diabetic rats, we analyzed the serum and fecal BA profiles across the three groups. Compared with the CON group, the serum and fecal BA levels of CA, TCA, CDCA, GCDCA, TDCA, and 7-KLCA were significantly higher in the TAC group, while only HDCA levels were significantly lower. After HDCA intervention, serum and fecal levels of HDCA, HCA, and THDCA were significantly elevated, while only CA levels were significantly reduced (Figures 5A,B). In addition, PBA, the PBA/SBA ratio, 12-hydroxy BAs (12-OH BAs), and the 12-OH/Non-12-OH BAs ratio were significantly higher in the serum and feces of the TAC group compared to the CON group. Following HDCA intervention, serum levels of SBA and Non-12-OH BAs were significantly increased, while PBA levels, the PBA/SBA ratio, and the 12-OH/Non-12-OH BAs ratio were significantly decreased. Fecal levels of TBA and Non-12-OH BAs were significantly increased, while 12-OH BAs and the 12-OH/Non-12-OH BAs ratio were significantly reduced (Figures 5C–F).

### 3.5 Effect of hyodeoxycholic acid on ileal protein expression in tacrolimus-induced diabetic rats

**FIGURE 6:**
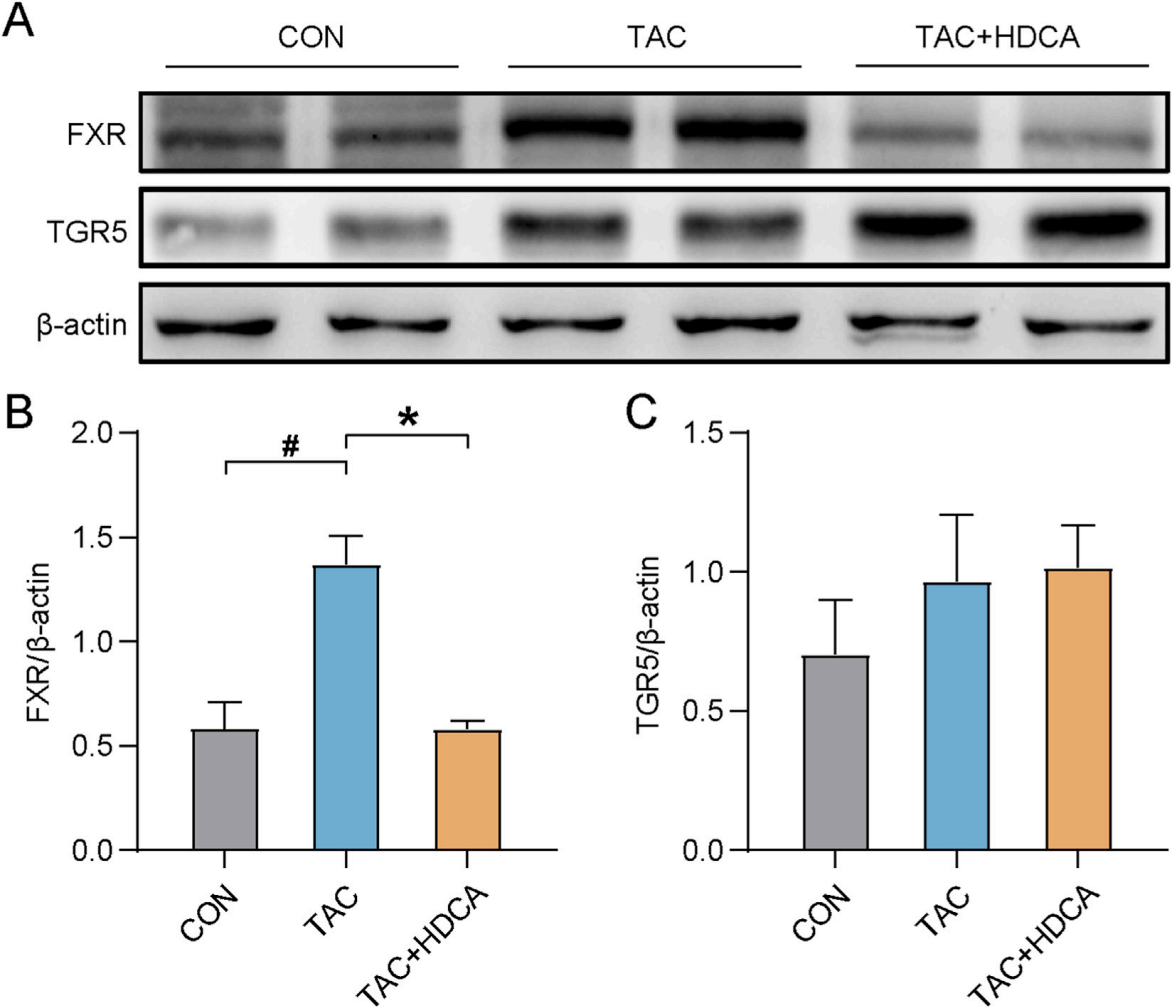
Effects of HDCA on the expression of FXR and TGR5 protein in ileum of TAC-induced diabetic rats. **(A)** Representative Western blot images of FXR, TGR5, and β-actin. **(B,C)** Densitometric analysis of FXR **(B)** and TGR5 **(C)** protein expression normalized to β-actin. Data are presented as the mean ± SD. Statistical significance was determined by ANOVA test followed by Tukey’s *post hoc* test (n = 6 per group). Significant differences are denoted as ^#^ *P* < 0.05, ^##^ *P* < 0.01, and ^###^ *P* < 0.001 for TAC versus CON group, and as ^*^ *P* < 0.05, ^**^ *P* < 0.01, and ^***^ *P* < 0.001 for TAC + HDCA versus TAC group.

Ileal FXR protein expression was upregulated in the TAC group compared with the CON group and reversed by HDCA (Figures 6A,B). Although TAC administration led to an increasing trend in TGR5 protein expression, the difference was not statistically significant. In contrast, TGR5 protein expression levels were significantly elevated after TAC and HDCA co-administration (Figures 6A,C).

### 3.6 Effect of hyodeoxycholic acid on ileal hormone secretion in tacrolimus-induced diabetic rats

**FIGURE 7:**
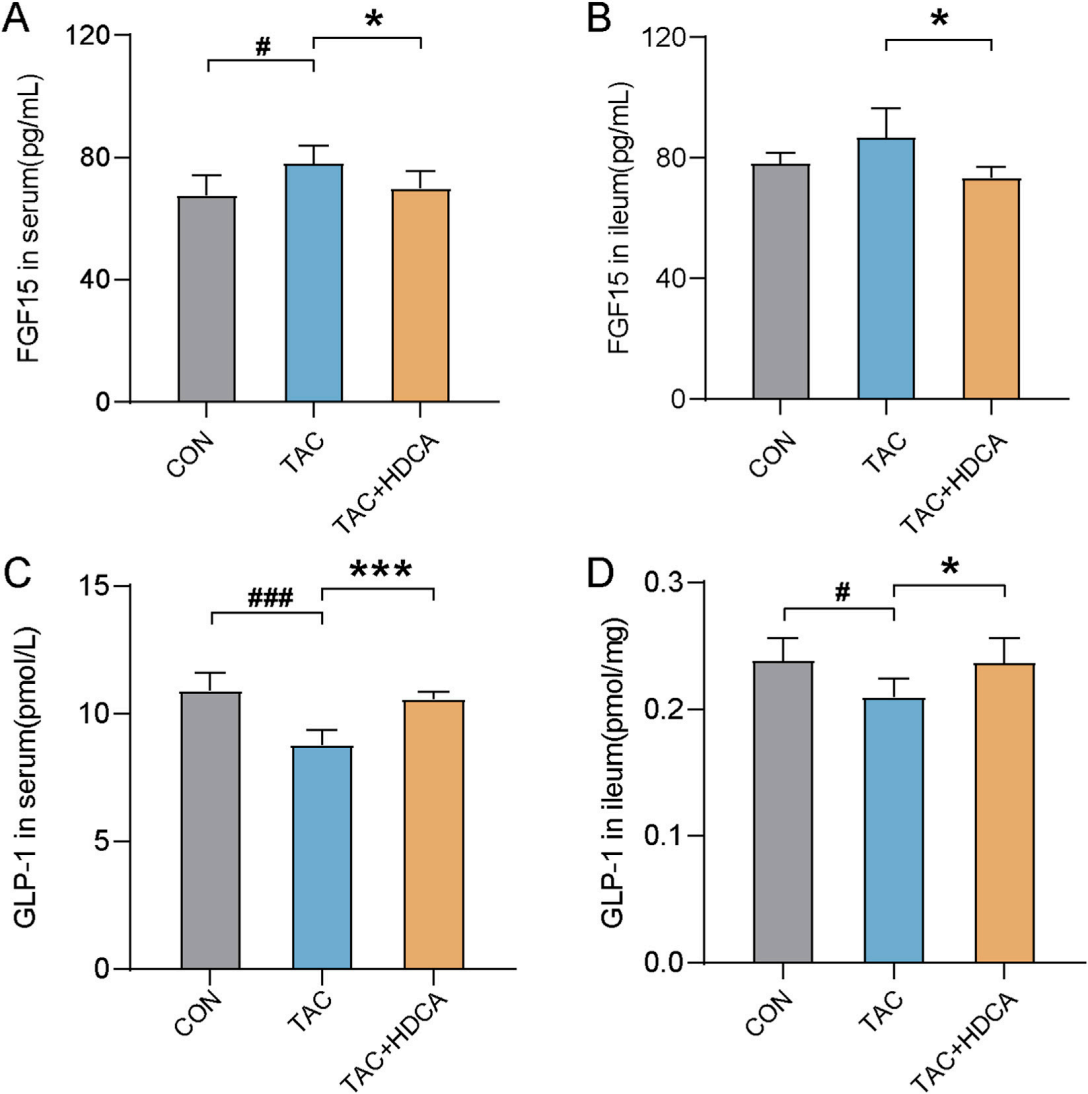
Effects of HDCA on ileal hormone secretion in TAC-induced diabetic rats. **(A)** Serum GLP-1 concentration. **(B)** Ileal GLP-1 concentration. **(C)** Serum FGF15 concentration. **(D)** Ileal FGF15 concentration. Data are presented as the mean ± SD. Statistical significance was determined by ANOVA test followed by Tukey’s *post hoc* test (n = 6 per group). Significant differences are denoted as ^#^ *P* < 0.05, ^##^ *P* < 0.01, and ^###^ *P* < 0.001 for TAC versus CON group, and as ^*^ *P* < 0.05, ^**^ *P* < 0.01, and ^***^ *P* < 0.001 for TAC + HDCA versus TAC group.

Activation of FXR in enteroendocrine cells leads to reduced GLP-1 release, which impairs pancreatic β-cell function and increases insulin resistance. Subsequently, we examined GLP-1 levels in serum and ileum. TAC administration significantly reduced serum and ileal GLP-1 levels compared with the CON group, whereas co-administration of TAC with HDCA enhanced serum and ileal GLP-1 secretion (Figures 7A,B). Binding of BAs to ileal FXR induced the expression of FGF15. ELISA results showed that TAC administration significantly increased serum FGF15 levels and elevated ileal FGF15 compared to the CON group, but no significant difference was observed. In contrast, FGF15 levels in serum and ileum were significantly reduced after co-administration of TAC with HDCA (Figures 7C,D). Accumulation of ileal HDCA may enhance GLP-1 secretion and directly downregulate intestinal FXR-FGF15 signaling.

### 3.7 Effect of hyodeoxycholic acid on hepatic BA synthase and gluconeogenesis gene expression in tacrolimus-induced diabetic rats

**FIGURE 8:**
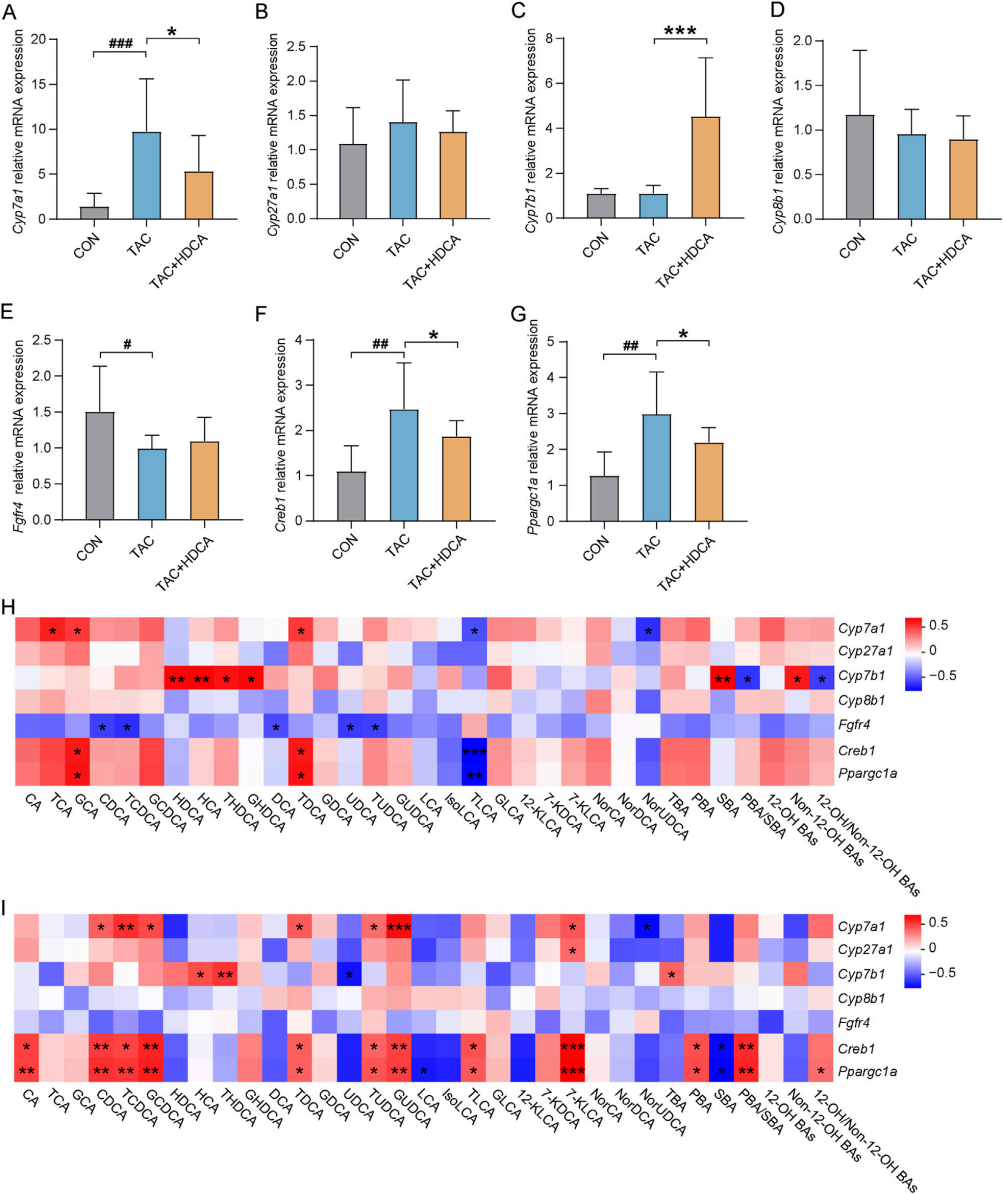
Effects of HDCA on hepatic gene expression and its associations with bile acid profiles in tacrolimus-induced diabetic rats. **(A–G)** Relative mRNA expression of genes involved in hepatic bile acid synthesis: *Cyp7a1* **(A)**, *Cyp27a1* **(B)**, *Cyp7b1* **(C)**, *Cyp8b1* **(D)**; and genes involved in hepatic gluconeogenesis regulation: *Fgfr4* **(E)**, *Creb1* **(F)**, *Ppargc1a* **(G)**. **(H,I)** Spearman correlation heatmaps showing the associations between hepatic gene expression and serum **(H)** and fecal **(I)** bile acid parameters, including individual bile acid concentrations, TBA, PBA, SBA, 12-OH and non-12-OH BAs, and their corresponding ratios. The color gradient represents the correlation coefficient, where red indicates a positive correlation and blue indicates a negative correlation. Color intensity reflects the strength of the association. Data are presented as the mean ± SD. Statistical significance was determined by ANOVA test followed by Tukey’s *post hoc* test (n = 6 per group). Significant differences are denoted as ^#^ *P* < 0.05, ^##^ *P* < 0.01, and ^###^ *P* < 0.001 for TAC versus CON, and as ^*^ *P* < 0.05, ^**^ *P* < 0.01, and ^***^ *P* < 0.001 for TAC + HDCA versus TAC group.

In the classical BA synthesis pathway, cholesterol is synthesized into two primary BAs, CA and CDCA, through a series of reactions including hydroxylation, isomerization, steroid side-chain oxidation, and cleavage in the presence of rate-limiting enzymes such as CYP7A1 and CYP8B1. In the alternative pathway, CYP27A1 and CYP7B1 and other metabolic enzymes synthesize CDCA (45, 46). To further elucidate the effects of HDCA on BA metabolism, we examined the gene expression levels of BA-related metabolic enzymes in the liver. RT-PCR results showed that the mRNA expression of *Cyp7a1* was significantly elevated after TAC administration compared to the CON group, and was reduced with HDCA co-administration (Figure 8A). In contrast, the expression of *Cyp7b1* was significantly increased in the TAC + HDCA group compared to the TAC group (Figure 8C). Additionally, the expression levels of the genes *Cyp27a1* and *Cyp8b1* remained unaltered by either TAC or HDCA treatments (Figures 8B,D).

To further elucidate whether the inhibition of the FXR-FGF15 axis contributes to the improvement of glucose homeostasis in HDCA-treated rats, we examined the expression levels of genes involved in hepatic gluconeogenesis. Compared with the CON group, TAC administration significantly reduced hepatic *Fgf4r* mRNA expression levels (Figure 8E) and significantly elevated *Creb1* and *Ppargc1a* mRNA expression levels. Notably, HDCA co-administration reversed the TAC-induced upregulation of *Creb1* and *Ppargc1a* (Figures 8F,G).

To elucidate the crosstalk between host BA regulatory networks and compartmentalized BA pools, we performed a correlation analysis between hepatic gene expression and BA profiles in both serum and feces. In the systemic circulation, HDCA and its structurally related derivatives emerged as key correlates with the alternative BA synthesis pathway. Specifically, the expression of *Cyp7b1* demonstrated a prominent and significant positive correlation with serum levels of HDCA, as well as HCA and THDCA. This specific *Cyp7b1*-HDCA axis contrasts with other circulating BAs, such as TLCA, which exhibited a strong negative correlation with the upstream regulators *Creb1* and *Ppargc1a* (Figure 8H). In the intestinal lumen, the correlation pattern underwent a shift. While serum HDCA was specifically linked to *Cyp7b1*, fecal HDCA, along with a broad spectrum of other microbiota-modified secondary BAs (e.g., TUDCA, GUDCA), showed widespread positive correlations with an expanded network of host genes, notably including the classical pathway enzyme *Cyp7a1*, *Creb1*, and *Ppargc1a* (Figure 8I). Collectively, these distinct correlation profiles indicate that hepatic gene expression, particularly *Cyp7b1*, is specifically coupled with systemic HDCA signaling, while simultaneously reflecting a broader synchronization with the luminal excretion of HDCA and other microbially derived BAs.

## 4 Discussion

Antibiotic-induced microbiome depletion is commonly used to study the role of the gut microbiome in pathological conditions (Sampson et al., 2016). A recent study found that the use of mixed antibiotics exacerbated the effects of TAC on gut microbiota composition and functional categories, which were significantly associated with glucose tolerance in mice (Han et al., 2019). In our previous study, we demonstrated that TAC induces diabetes mellitus and that alterations in the composition of the gut microbiota (decreased abundance of *Akkermansia* and *Ruminococcus* and increased abundance of *Enterococcus* and *Bifidobacterium*) were significantly correlated with some amino acids and BAs in the metabolites (Jiang et al., 2024). To assess the role of gut microbiota in the progression of TAC-induced metabolic disorders, the present study was conducted in rats administered TAC and mixed antibiotics by gavage daily for 10 consecutive weeks. The results showed that FBG, OGTT-AUC, and HOMA-IR indices further increased with antibiotic intervention, while HE staining and immunohistochemistry revealed further aggravation of TAC-induced abnormalities in glucose tolerance and pancreatic islet cell damage. Meanwhile, antibiotics significantly enhanced TAC-induced alterations in the microbial composition of rats, and sequencing results showed a significant decrease in the relative abundance of gut bacterial genera such as *Lactobacillus*, *Bifidobacterium*, and *Romboutsia*. Derived enzymes from the microbiome in the gut mediate the complex biotransformation of BAs produced in the liver (Ussar et al., 2015), and the deconjugation of bile acids is catalyzed by bile salt hydrolase (BSH), an enzyme widely expressed by various gut bacteria, prominently including *Lactobacillus*, *Bifidobacterium*, *Clostridium*, and *Bacteroides* (Foley et al., 2019). Antibiotics affect BSH, which is involved in the transformation of SBA, inhibit SBA formation in the intestine, and increase bound PBA in plasma, liver, and intestine in mice (Zhang et al., 2014). We analyzed serum BAs and found that antibiotic-induced depletion of gut flora altered the size and composition of the BA pool, with significant reductions in serum TBA, PBA, and SBA in fasted rats serum. Further quantification revealed particularly pronounced decreases in HDCA and NorUDCA following antibiotic intervention. These results suggest that antibiotic-mediated depletion of gut flora exacerbates TAC-induced disruption of glucose metabolism, alteration of the gut microbiome, and the composition of the BA pool in rats.

HDCA, a key BA in porcine BAs and their derivatives, has been demonstrated to exert cholesterol-lowering and glucose homeostasis-modulating effect (Kuang et al., 2023; Zheng et al., 2021a). In the present study, we employed HDCA (100 mg/kg/day) to intervene in TAC-induced diabetic rats and found that it improved glucose tolerance abnormalities and insulin resistance, while also enhancing insulin sensitivity to some extent. The HDCA dosage was selected based on previous study demonstrating that intragastric administration of 100 mg/kg/day HDCA significantly improved non-alcoholic liver disease in C57 mice, while increasing the dose to 150 mg/kg/day did not further enhance the efficacy (Kuang et al., 2023). Additionally, we observed that TAC significantly elevated serum and fecal BA levels (e.g., CA and CDCA) synthesized via the classical pathway. Following HDCA intervention, the serum and fecal levels of these BAs were significantly reduced. Although the precise mechanism of HDCA remains to be fully elucidated, our data suggest that its beneficial effects are likely mediated through the regulation of BA-metabolizing enzymes and related signaling pathways. BAs act as key metabolic integrators in regulating fat, glucose, and energy metabolism through gene expression modulation. This signaling may be partially dependent on, or independent of, the FXR signaling pathway and involves the TGR5 receptor (Lefebvre et al., 2009). FXR is primarily located in the liver and intestine, where it serves a crucial role in BA metabolism (Clifford et al., 2021). In our study, we found that TAC upregulated ileal FXR protein expression, whereas HDCA intervention reversed this trend. In contrast, neither TAC nor HDCA affected ileal TGR5 protein expression. FGF15/19 (FGF15 in rodents and FGF19 in humans) is an endocrine hormone secreted from the distal ileum, and its production is tightly regulated by ileal FXR activity, which is influenced by varying BA species (Di Ciaula et al., 2022). FXR receptors in the distal ileum regulate the synthesis and secretion of FGF15/19, which is then transported to the liver via the enterohepatic circulation. In the liver, FGF15 binds to fibroblast growth factor receptor 4 (FGFR4). This FGF15/19-FGFR4 signaling axis activates downstream pathways that suppress hepatic CYP7A1 expression, thereby reducing *de novo* bile acid synthesis and maintaining bile acid homeostasis (Bozadjieva-Kramer et al., 2024; Katafuchi and Makishima, 2022). Moreover, in enteroendocrine cells, BAs-activated FXR disrupts GLP-1 secretion and glucagonogen expression. The FXR/GLP-1 axis represents a potential novel mechanism through which BAs modulate glucose metabolism (Trabelsi et al., 2015). Our findings demonstrated that TAC decreased serum and ileal GLP-1 levels, which were subsequently reversed following HDCA intervention. Activation of FXR signaling in the intestine may inhibit GLP-1 secretion, whereas HDCA appears to contribute to the restoration of glucose homeostasis by suppressing intestinal FXR protein expression and thereby promoting GLP-1 secretion in enteroendocrine cells.

The classical pathway of BA synthesis is initiated by CYP7A1 and further regulated by CYP8B1, whereas the alternative pathway is initiated by CYP27A1 and involves CYP7B1 (Fleishman et al., 2024; Pandak et al., 2019). It has been shown that HCA and its derivatives (such as HDCA) are Non-12-OH BAs produced via the alternative pathway, where CYP8B1 is the key enzyme for synthesizing CA, a 12-OH BA, and CYP7B1 is responsible for producing Non-12-OH BAs (e.g., CDCA, UDCA, HCA, and their conjugates). An increased proportion of Non-12-OH BAs, often associated with activation of the alternative bile acid synthesis pathway, has been linked to improved glucose and lipid metabolism (Haeusler et al., 2013; Jia et al., 2021). The depletion and downregulation of CYP8B1, alongside the upregulation of CYP7B1, play beneficial roles in host metabolic processes (Worthmann et al., 2017). In our study, we found that TAC upregulated *Cyp7a1* mRNA expression levels and increased the levels of BAs synthesized via the classical pathway, such as CA and CDCA, in both serum and feces. HDCA intervention significantly reduced *Cyp7a1* expression levels and downregulated the serum and fecal levels of these classical BAs. These findings suggest that TAC may increase BA synthesis and the BA pool size by upregulating *Cyp7a1* expression, while HDCA intervention may inhibit the classical pathway of BA synthesis. Moreover, HDCA intervention significantly upregulated *Cyp7b1* expression levels and notably reduced the ratio of 12-OH to Non-12-OH BAs in serum and feces. These results suggest that HDCA may increase the proportion of Non-12-OH BAs by activating the BA replacement pathway. A recent study found that HDCA treatment downregulated hepatic *Cyp7a1* expression and upregulated *Cyp7b1* expression, which aligns with our findings. Furthermore, the therapeutic effects of HDCA were diminished following hepatic *Cyp7b1* knockdown, whereas overexpression of *Cyp7b1* significantly alleviated metabolic disorders (Kuang et al., 2023), indicating that the alternative pathway of CYP7B1-driven BA synthesis may play a role in ameliorating HDCA-induced TAC diabetes.

**FIGURE 9:**
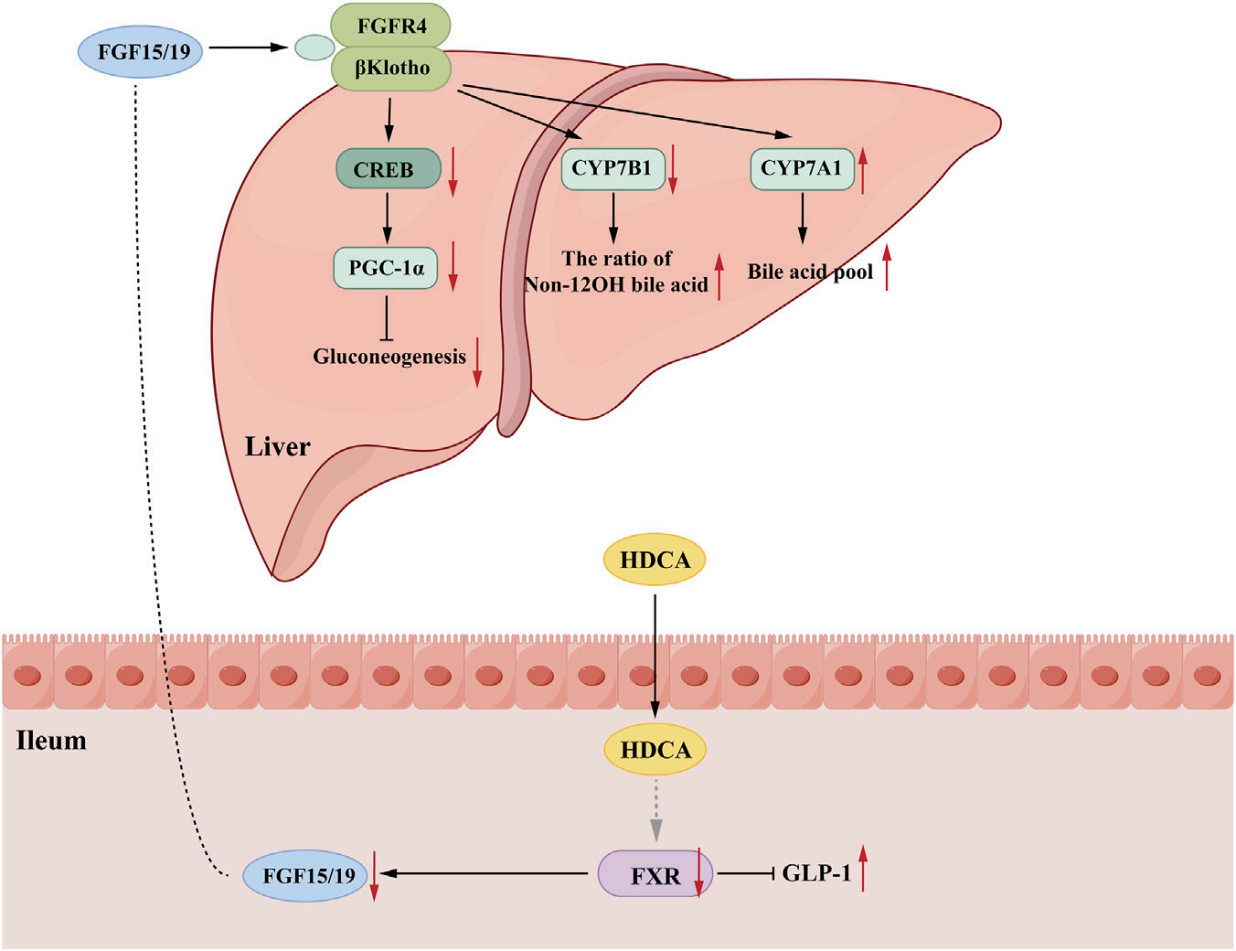
Potential mechanisms through which HDCA contributes to the amelioration of TAC-induced glucose metabolism disorders.

FGF15 overexpression enhances insulin sensitivity and protects against diet-induced obesity in mice, highlighting its role in metabolic regulation (Fu et al., 2004; Katafuchi et al., 2022). FXR activation in the intestine induces FGF15/19 expression, after which the hormone enters the portal vein, mediated by FGFR, a tyrosine kinase receptor, and the KLB coreceptor (beta-Klotho). This receptor complex exerts an inhibitory effect on gluconeogenesis and promotes liver glycogen synthesis (Kliewer et al., 2015). The complex can inhibit gluconeogenesis-related gene expression through the CREB-PGC-1α pathway (Potthoff et al., 2011). Inhibition of ileal FXR-FGF15 signaling has been associated with improvements in metabolic disorders, including NAFLD, obesity, and insulin resistance (Jiang et al., 2015; Katafuchi et al., 2022). Furthermore, HDCA has been identified as a naturally occurring FXR antagonist, and it has been demonstrated that HCA inhibits the intestinal FXR-FGF15/19 signaling pathway (Zheng et al., 2021a). Our results showed that FGF15 expression levels were reduced in both serum and ileum, while hepatic *Fgf4r* mRNA expression levels were elevated following HDCA intervention. These findings suggest that HDCA inhibits the enterohepatic FXR-FGF15 axis. Hepatic BA-FXR signaling may regulate blood glucose levels by reducing gluconeogenesis and inducing glycogen synthesis (Shapiro et al., 2018). Upon activation of the hepatic FGFR4-βKlotho receptor complex, gut-derived FGF15 exerts potent insulin-like metabolic effects; it promotes hepatic glycogen synthesis while simultaneously suppressing gluconeogenesis, primarily through the targeted inhibition of the CREB-PGC-1α pathway (Katafuchi and Makishima, 2022; Potthoff et al., 2011). In our study, HDCA intervention reduced the mRNA expression levels of *Creb1* and *Ppargc1a*, suggesting that HDCA may ameliorate TAC-induced glucose metabolism disorders by reducing hepatic gluconeogenesis (Figure 9).

This study has several limitations. First, due to experimental constraints and the scope of the present study, pharmacological or genetic intervention models (e.g., FXR agonists/antagonists or FXR-deficient models) were not included. Therefore, the causal role of the FXR-FGF15 signaling axis in mediating the beneficial metabolic effects of HDCA could not be fully established. Second, the study lacked dose-response evaluation, comprehensive toxicological assessment, and an HDCA-only control group, limiting a thorough evaluation of the optimal dosage and the direct metabolic effects of HDCA. In addition, mechanistic interpretations were primarily based on mRNA expression data without validation at the protein or enzyme activity levels. Furthermore, the targeted LC-MS/MS bile acid profiling method used in this study did not include glucuronidated bile acids, such as HDCA-6-glucuronide and HDCA-24-glucuronide, because reliable quantification depends on authentic reference standards and validated analytical methods, while the availability of these analytical methods and reference standards remains limited. Although antibiotic treatment suggested a potential involvement of the gut microbiota in HDCA-mediated metabolic regulation, the current evidence remains indirect, and microbiome analyses were not performed consistently across all experiments. Future studies incorporating dose-response designs, protein-level and functional validation, germ-free animal models, fecal microbiota transplantation, and dedicated analytical methods for glucuronidated bile acids will help elucidate the microbiota-dependent mechanisms underlying the metabolic effects of HDCA and provide a more comprehensive understanding of phase II bile acid metabolism. In conclusion, the present study demonstrates that HDCA ameliorates TAC-induced glucose metabolic disorders, likely through suppression of the enterohepatic FXR-FGF15 signaling axis.

## 5 Conclusion

In conclusion, this study demonstrates that antibiotic-induced microbiome depletion worsens serum BA alterations in TAC-induced diabetic rats. HDCA treatment significantly ameliorates TAC-induced glucose metabolism disorders and may influence BA synthesis and the BA pool size by downregulating hepatic BA synthase CYP7A1 and upregulating the expression of CYP7B1. Furthermore, HDCA may affect liver function by inhibiting intestinal FXR protein expression, thereby increasing GLP-1 secretion in enteroendocrine cells, and by disrupting the enterohepatic FXR-FGF15 axis, which in turn affects liver gluconeogenesis, thus contributing to improved glycemic homeostasis.

## Data Availability

The original contributions presented in the study are publicly available. The 16S rRNA sequencing datasets have been deposited in the NCBI Sequence Read Archive (SRA) under BioProject accession PRJNA1497351.
